# Intra and Inter-Rater Reliability of a Novel Isometric Test of Neck Strength

**DOI:** 10.3390/sports11010002

**Published:** 2022-12-21

**Authors:** Lesley McBride, Rob S. James, Siân Alsop, Samuel W. Oxford

**Affiliations:** 1 School of Nursing, Midwifery and Health, Coventry University, Priory Street, Coventry CV1 5FB, UK; 2 Faculty of Life Sciences, University of Bradford, Bradford BD7 1DP, UK; 3 Research Centre for Global Learning, Coventry University, Priory Street, Coventry CV1 5FB, UK; 4 Centre for Sport, Exercise and Life Sciences, Institute for Health and Wellbeing, Coventry University, Priory Street, Coventry CV1 5FB, UK

**Keywords:** cervical spine, neck strength, muscle testing, test-retest, profiling, peak force

## Abstract

There is no single, universally accepted method of measuring isometric neck strength to inform exercise prescription and injury risk prediction. This study aimed to establish the inter- and intra-rater reliability of a commercially available fixed frame dynamometer in measuring peak isometric neck strength. A convenience sample of male (n = 16) and female (n = 20) university students performed maximal isometric contractions for flexion (Flex), extension (Ext), left- (LSF) and right-side flexion (RSF) in a quadruped position over three sessions. The intra-rater reliability results were good-to-excellent for both males (ICC = 0.83–0.90) and females (ICC = 0.86–0.94) and acceptable (CV < 15%) across all directions for both males and females. The inter-rater reliability results were excellent (ICC = 0.96–0.97) and acceptable (CV < 11.1%) across all directions. Findings demonstrated a significant effect for sex (*p* ≤ 0.05): males were stronger in all four directions, and a significant effect for direction (*p* ≤ 0.05): Ext tested stronger (193 N) than Flex (176 N), LSF (130 N) and RSF (125 N). The findings show that the VALD fixed frame dynamometer can reliably assess isometric neck strength and can provides reference values for healthy males and females.

## 1. Introduction

Maximal force-generating capabilities are commonly monitored in athletes and can be evaluated using dynamic or isometric muscle contractions [1,2]. The increased popularity of isometric tests to assess an athlete’s maximum strength and ability to exert maximal force in the shortest time possible means it is important to ensure the reliability of the data obtained to prescribe, monitor and alter training programmes [3]. To date the reliability of isometric muscle strength measurements has been demonstrated in multiple anatomical regions, especially lower extremities [3]. The importance of isometric neck strength in four directions (Flexion, Extension, Left and Right-side Flexion) and its association with head and neck kinematics [4] and sports related concussion has been demonstrated [5,6]. However, a lack of evidence exists for assessing neck strength [7,8], leaving practitioners without a reliable method for doing so. Moreover, the common usage of handheld dynamometers (HHDs) for measuring isometric strength in sporting and clinical contexts [8] is, arguably, problematic. These devices do offer a useful portable and affordable option for measuring isometric neck strength in sagittal (flexion/extension) and frontal (side flexion) planes [9]. However, usage is affected by variable factors such as tester strength and device stabilization [10], which can undermine reliability and validity. Devices that test neck strength eccentrically with a “break” test rather than concentrically with a “make” test [11] pose additional concerns.

Fixed frame dynamometers (FFDs) offer another option for measuring neck strength. FFDs are considered the gold standard for isometric neck muscle strength quantification [12,13]. However, general inconsistencies in methodological and testing protocols often render assessment repeatability difficult, and the cross-examination of normative reference values impossible [14,15]. These devices can measure isometric strength without tester-provided resistance during measurement (a limitation in reliability tests of HHDs [3]), but the testing position varies; much non-sporting neck strength research has been performed in the seated position with the torso fixed by seat belts [8]. This position may have specific sporting relevance (e.g., in motor sport), but it raises questions about effective muscle isolation due to bracing against restraints.Importantly, it is not transferable to sports where many injuries occur when the body is unrestrained and in the horizontal position or whilst running, for example as in rugby [16].

The relative reliability (Inter class correlation coefficients ICC) of neck strength testing in the four directions using HHD (ICC = 0.77–0.9) [7] MHHD (mounted HHD) (ICC = 0.77–0.92) [7] and FFD (ICC = 0.86–0.94) [16] has proven similar. However, absolute reliability (Coefficient of Variation CV) has previously been under reported, making comparisons across methodologies challenging for practitioners [8]. Research using FFDs [16] employs bespoke laboratory-based equipment, making it unsuitable for adoption by practitioners. The equipment’s lack of commercial availability, specificity of testing to the research context and body position, and small participant cohort limit the generalizability of conclusions drawn. Although the study undoubtedly progresses understanding of neck strength, the bespoke nature of the testing equipment makes wider adoption unviable, limiting its potential impact in a broader context. Furthermore, the test position adopted was devised specifically for rugby union players who participate in a scrum (i.e., forwards), as torso bracing against a bench was allowed. This choice challenges ecological validity because, as in the case of adopting a seated position, torso bracing potentially makes the force produced by the neck muscles difficult to isolate from the bracing force production. A potential alternative would be to assess isometric neck strength in a similar, but unsupported, pose such as the quadruped position. Although this position is not uncommon (e.g., it was adopted in the study by Hall et al. [17]), it potentially limits generalizability to most sports which do not involve torso support. Such studies test bespoke equipment that is reliable but not commercially available, and therefore specific to the research setting.

Even commercially available equipment specifically designed to measure neck strength, such as the Multi-Cervical Unit (MCU) (BTE, Birmingham), presents barriers to testing accuracy. The MCU’s load cells are limited to 50 lbs (22.6 kg; 222 N), which is insufficient for stronger athletes like rugby players who regularly record force readings of greater than 400 N [9,18]. Additionally, this device is not portable, significantly limiting its usability. The VALD ForceFrame (Newstead, Australia) is one of several commercially available FFDs that are portable and lightweight. It has been promoted for the assessment of force during isometric muscle contractions of lower and upper limbs. The load cell sensors have a maximum capacity of 1000 N, a safe overload value of 1500 N, and a maximum overload per sensor of 2000 N. The resolution is 1 N and the sample rate 400 Hz. The ForceFrame has produced an excellent ICC score of 0.94 in adductor groin strength assessment in professional Australian footballers [3]. No reliability data, however, is currently available for isometric neck strength assessment in flexion (Flex), extension (Ext) and left- and right-side flexion (LSF and RSF). Therefore, the usage and accessibility advantages of many FFDs are undermined by issues of reliability [10], or lack of available data for assessing specific types of neck strength.

The current study responds to the need to provide reliable neck strength testing data using available equipment in a verified testing position. Reliability refers to the consistency of a test or measure [19]; a measure is considered to have high test-retest reliability if it produces comparable results under consistent conditions over time [20]. The aims of this study were 1: to examine the test-retest and inter-rater reliability of the VALD ForceFrame for assessing isometric neck strength in four directions in a quadruped position, and 2: to compare male and female differences in a sample of healthy participants. This sample was chosen to test reliability in both sexes as it is well known that the morphology of the neck differs between men and women [21]. The utilization of this test position, which is advocated by VALD, reduces the variability introduced when additional testing equipment is required, such as a chair, and is reproducible by each individual participant between tests.

## 2. Materials and Methods

A double-session repeated measures intra-rater and inter-rater reliability study was performed.

### 2.1. Subjects

Ethical approval was provided by Coventry University Human Research Ethics Committee. Participants were informed about the study and gave written informed consent prior to participation. A convenience sample of n = 40 participants (n = 20 males and n = 20 females) were recruited with a required sample size of n = 18 based on a priori power analysis (effect size *f* = 0.8, α = 0.05 and β = 0.02) (G*Power). Two male participants were unable to attend for the second measurement session and two male participants’ data was excluded as the repeat measurements were more than 3 standard deviations from the mean [18]. All participants were aged 18 years or over, physically active, had no current neck pain or pathology and no previous neck injury (which was an exclusion criterion). Each participant visited the testing laboratory on two occasions, separated by at least 72 h. Upon arrival, measurements of height (to the nearest 0.5 cm), body mass (to the nearest 0.5 kg), and neck girth (to the nearest 0.5 cm) measured immediately cranial to the thyroid cartilage with the participant instructed to look straight ahead, were recorded (Table 1).

### 2.2. Procedure

Each participant completed an isometric warmup, pushing their head against their own hand in each of the four test directions (Flex, Ext, LSF and RSF) with progressively increasing force from 50% to 75% of their self-perceived maximal effort. This was repeated a further four times for each direction with a ten second rest between contractions.

**Figure 1:**
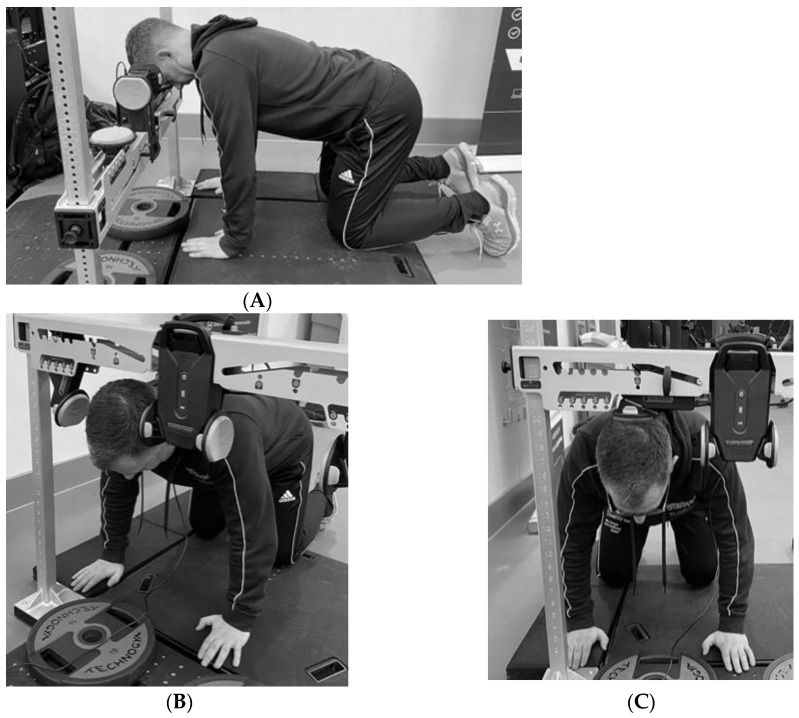
Test position adopted for (**A**) = Flexion, (**B**) = Right side flexion and (**C**) = Extension for the assessment of isometric neck strength in the VALD ForceFrame (Generation 1) testing rig.

Testing was carried out by Tester 1 (trial 1), a physiotherapist who instructed participants to assume the quadruped (start) position: hands shoulder width apart perpendicularly below the proximal joint, scapulae drawn together, elbows fully extended, and hips and knees set at 90 degrees (Figure 1).

**Figure 2:**
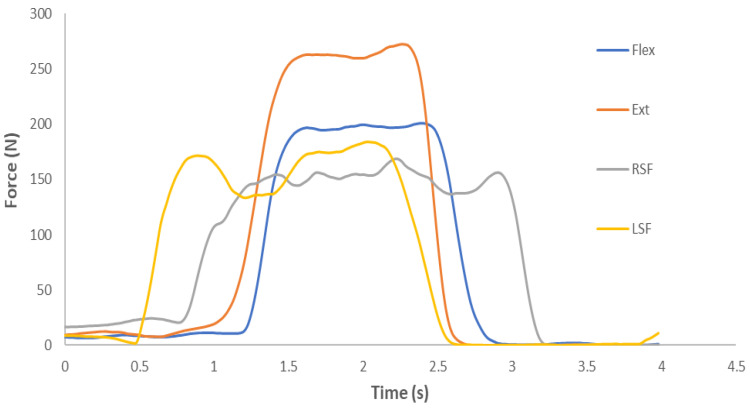
0 Example of the force time curve for Flexion (Flex), Extension (Ext), Left-side flexion (LSF) and Right-side Flexion (RSF) for a male participant.

The load cell was in contact with the frontal bone superior to the eyebrows for Flex; the occiput for Ext; and the temporal bone just above the superior aspect of the ear helix for LSF and RSF. The direction of testing was randomly ordered. Pre-test, participants became familiar with pushing against the load cell at an estimated 80% maximum voluntary isometric contraction (MVIC). The VALD ForceFrame was zeroed between each test. Participants were instructed to inhale/exhale and, when ready, push maximally for approximately three seconds [16]. Verbal encouragement was provided [3] and a minimum of ten seconds were allowed between each of the three repetitions. The time between test positions was three minutes. The peak force from the three repetitions in each of the four directions was selected as the MVIC (Figure 2). MVICs were transferred to a computer through USB connection and analysed using Microsoft Excel. On the second laboratory visit (trial 2) intra-rater reliability assessment was completed on n = 38 participants. In addition, inter-rater reliability was assessed by measuring participants (n = 20) twice, in a random order of the two testers, by Tester 1 (physiotherapist) and Tester 2 (strength and conditioning coach), with a minimum of 30 min between trials. Whilst usage of the force frame should reduce variability between testers compared to some previous methods, it is important to quantify any differences to justify usage of this proposed change in methodology.

### 2.3. Statistical Analysis

Statistical analysis was performed using SPSS and the criterion for statistical significance was set at *p* ≤ 0.05. Descriptive statistics (mean ± SD) were calculated for peak force (N) in each of the four directions. Two-way analysis of variance was used to compare peak isometric neck force between each of the four directions and sex, using direction and sex as fixed factors. Mauchly’s test of sphericity was used to determine if sphericity was violated and a Greenhouse-Geisser correction was used when this occurred. Where differences were noted in ANOVA, pairwise comparisons (Bonferroni adjusted) were made to identify where significant differences occurred. Effect size for the ANOVA statistics was estimated using partial Eta squared (η^2^_p_) for analysis of variance [22].

To determine the relative reliability of the measures, intraclass correlation coefficients ICC_(3,1)_ were calculated for the peak force values from the three trials for each of the four directions [19]. The CV was calculated based on the mean square error term of logarithmically transformed data. Acceptable reliability was then determined as an ICC_(3,1)_ > 0.70 and a CV of <15% [2].

Absolute reliability of the peak isometric force was determined using the standard error of measurement (SE_m_) calculated using the formula SE_m_ = SD × √ (1 − ICC) where SD value was the combined SD value from the two trials and the ICC values were the two-way mixed model single measure of consistency [20]. The minimal detectable change (MDC) was determined using the formula MDC = 1.96 × √2 × SE_m_ [23].

## 3. Results

There was a significant difference between sexes for: height t (36) = 6.28, *p* = 0.001; body mass t (36) = 4.70, *p* = 0.001; and neck girth t (36) = 11.2, *p* = 0.001 (Table 1).

Inter-rater reliability data showed an ICC of 0.96 (CV 11.1%) for Ext, 0.97 (CV 7.6%) for Flex, 0.97 (CV 10.7%) for RSF and 0.97 (CV 9.7%) for LSF, which indicated excellent reliability. Intra-rater reliability results from trial 1 and trial 2 from the single measure ICCs were good to excellent across all directions ICC > 0.87 and a CV % < 14% for both males and females (Table 2). The highest SE_m_ was achieved in males during Ext (25 N) for the group indicating the highest level of variability in the four directions measured, whereas Flex female and RSF female (6 N), were the lowest (Table 2). When the MDC was compared with the overall mean for each direction, the following values were calculated to indicate that a meaningful change for clinical practice had occurred in neck strength: Ext: 34 N (female), 69 N (male); Flex: 16 N (female), 43 N (male); LSF: 27 N (female), 45 N (male); and RSF: 16 N (female), 54 N (male) (Table 2).

Analysis of variance for isometric neck strength showed a significant main effect for sex F(1, 31) = 92.1, *p* ≤ 0.001, η^2^_p_ 0.75. Over the four directions males produced greater MVICs than females: Ext 102%; Flex 80%; LSF 67% and RSF 70% (Table 2). There was a significant main effect for direction F(2.17, 67.1) = 103.62, *p* ≤ 0.001, η^2^_p_ 0.77 (Table 2). Post hoc comparisons indicated significant differences between: Flex and Ext (9.89%) (*p* = 0.023); Flex and LSF and RSF (35.6% and 40.4%) (*p* ≤ 0.001) respectively; Ext and LSF and RSF (49.0% and 54.2%) (*p* ≤ 0.001) respectively. There was no significant difference between LSF and RSF (*p* = 0.508). There was a significant interaction effect between sex and direction F(2.03, 62.91) = 24.99, *p* ≤ 0.001, η^2^_p_ 0.45.

## 4. Discussion

This study examined the reliability of an isometric, concentric “make” test performed using the VALD ForceFrame from a quadruped position for Flex, Ext, LSF and RSF in a sample of healthy males and females. To determine the reliability of a test, ICCs, CV and 95% CI should be calculated [24,25]. ICCs provide information about the consistency and agreement between two sets of data and the CV is an estimate of the measurement error. No previous acceptable reliability data for isometric neck strength has been fully reported in the literature. However, in the analysis of isometric mid-thigh pull (IMTP), a minimal acceptable threshold of ICC > 0.7 and CV < 15% [26] had been proposed and the values accepted. In the present study, for both male and females for each of the four directions, our intra-rater ICC ranged from 0.83 to 0.94 with CVs ranging from 5.2% to 14% (Table 2). The inter-rater reliability data ranged from 0.96 to 0.97 with a CV % ranging from 7.6% to 11.1%. This indicates that the protocol used in this study has good inter- and intra-rater reliability. Findings for the reliability assessment of isometric concentric neck strength are in line with those reported with a custom-made device (0.90 to 0.97) [16], and other commercially available FFD devices (0.96 to 0.99) [17] and (0.85 to 0.97) [27]. However, these studies have not reported CV % and CIs, rendering the level of reliability questionable as only ICC values were reported [26]. These findings have significant implications for the further development of research into neck strength and this is the first study to test the clinical utility of a commercially available fixed frame device for the measurement of isometric concentric neck strength. This work strengthens the argument for measuring isometric concentric neck strength using the VALD ForceFrame in a clinical and sporting context.

For absolute reliability, the SE_m_ and the MDC provide useful values to detect whether the change in an individual or group is ‘real’ and is not secondary to measurement error. Additionally, the MDC is of clinical importance as it indicates the extent to which an individual’s strength recording needs to change to be sure ‘real’ change, and not just measurement error, has occurred. The absolute reliability (SE_m_) for the group data was calculated for each direction and both sexes, which indicates the standard deviation expected in MVIC values when repeatedly testing a single individual. SE_m_ findings from the current study ranged from 5.63 (female RSF) to 24.81 (male Ext) and are similar to the previously reported SE_m_ values of Flex 19, Ext 16, LSF 16 and RSF 14 by Almosnino et al. [28] who utilized a custom-made device. The difference in values for extension may be explained by differences in start position adopted by the two studies. In this study a change greater than 34 N (females) or 69 N (males) for Ext, 16 N (female) or 43 N (males) for Flex, 28 N (females) or 45 N (males) for LSF, and 16 N (females) or 54 N (males) for RSF is required to indicate that a meaningful clinical change (MDC) had occurred in MVIC values. This is important to consider when using measurements to inform and measure effective training programmes.

The pattern of strength for all participants reported in this study aligns with that reported by previous studies [8]. The values obtained are consistent with previous data collected on neck strength in a seated and a simulated contact posture in rugby, with the largest maximal force produced in Ext [18,28] (Table 2). The male participants produced an average peak force of 269 N, which was similar to that previously recorded by healthy males in a similar, but torso-supported, testing position (234 N) [18], and in a seated position (252 N) [29]. Males were on average 102% stronger than females in Ext (Table 2). A similar pattern for male and female differences was reported across all directions: Flex (80%), RSF (70%) and LSF (67%). The larger forces recorded by males in this study are consistent with data recorded in the aforementioned studies and all other studies on neck strength included in the systematic review by Selistre [8]. The larger force produced by males can be attributed to anatomical differences between sexes regarding muscle morphology [30]. The larger forces in Ext for both sexes are perhaps in part due to the larger cross-sectional area of extensor muscles in relation to flexors, which plays an important role in neck and head postural stability [31]. Findings relating to differences in force produced, disaggregated by sex, offer practitioners valuable insight when establishing baseline neck strength measurements in different populations.

The testing procedure adopted in this study offers practitioners a simple protocol in comparison to existing options. The procedure was effective and showed high clinical applicability due to the low equipment burden for test completion [10,16,32]. The quadruped position adopted minimized the potential variable impact of external restraints on testing [33]. In the quadruped position, stability was achieved by requiring participants to retract their scapulae fully to engage their thoracic muscles, enabling a standardized, stable and highly reproducible test position [16]. This position will be relevant to various sports which involve free, unrestrained body postures.

The findings of this study are applicable to the tested participant population. This is a limitation which should be taken into consideration when comparing the findings to other populations, or other methods used to assess isometric concentric neck strength.

## 5. Conclusions

Neck strength is considered a key protective feature across sports. The VALD ForceFrame provides a reliable measure for maximal isometric concentric strength for the neck flexor, extensor and lateral flexor muscles when assessed in a quadruped test position in a population of healthy males and females. The reliability of both the equipment and protocol facilitates increased tester confidence in, and accurate cross-population comparison of, neck health measurement. Findings support current drives to better understand links between a strong neck and injury mitigation. The usable, clinically applicable and commercially available protocols presented offer a gold standard that advances positive change in the discipline.

## Figures and Tables

**Table 1:** Anthropometric data for study participants (n = 36) (mean ± SD).

	n	Age	Height (cm)	Mass (kg)	Neck Girth (cm)
Males	16	23.1 ± 4.7	180 ± 8	86.5 ± 11.6	40.7 ± 2.2
Females	20	24.5 ± 8.2	165 ± 6 ^a^	65.0 ± 12.9 ^a^	33.9 ± 2.2 ^a^
Total	36	23.8 ± 6.6	173 ± 10	75.8 ± 16.3	37.3 ± 4.1

^a^ Significantly different to males (*p* < 0.05).

**Table 2:** Mean ± SD values and CV % for male (n = 16) and female (n = 20) maximum isometric neck force and intra-rater reliability values for all 4 directions.

					ICC				CV	
Group	Direction	Trial 1 (N)	Trial 2 (N)	Total (N)	ICC_(3,1)_	95% CI	SEm (N)	MDC (N)	(%)	95% CI
Female	Ext	131 ± 37	135 ± 30	133 ± 34 ^a^	0.87	0.69–0.94	12	34	10.4	7.8–15.6
Male		270 ± 77	268 ± 62	269 ± 69	0.87	0.66–0.95	25	69	11.1	8.1–17.7
Total		193 ± 90	194 ± 81	193 ± 85						
Female	Flex	127 ± 22	134 ± 19	130 ± 21 ^a^	0.92	0.81–0.97	6	16	5.2	3.9–7.7
Male		231 ± 47	239 ± 39	235 ± 43	0.87	0.68–0.95	15	43	6.8	5.0–10.8
Total		173 ± 63	180 ± 61	176 ± 61 ^b^						
Female	LSF	95 ± 29	105 ± 26	100 ± 26 ^a^	0.86	0.69–0.94	10	27	11.0	8.2–16.4
Male		156 ± 55	177 ± 47	167 ± 51	0.90	0.74–0.96	16	45	10.5	7.6–16.6
Total		123 ± 51	137 ± 51	130 ± 51 ^b,c^						
Female	RSF	90 ± 21	102 ± 24	96 ± 23 ^a^	0.94	0.85–0.97	6	16	8.3	6.2–12.3
Male		159 ± 53	166 ± 43	163 ± 47	0.83	0.58–0.94	20	54	14.0	10.2–22.5
Total		121 ± 52	130 ± 46	125 ± 49 ^b,c^						

ICC = intraclass correlation coefficient; 95% confidence interval for the ICC_(3,1)_ single measure; SE_m_ = standard error of measurement; MDC = minimal detectable change; Ext = extension; Flex = flexion; LSF = left-side flexion; RSF = right-side flexion. ^a^ Significant difference between males and females *p* ≤ 0.05. ^b^ Significant difference between Ext with Flex, LSF and RSF *p* ≤ 0.05. ^c^ Significant difference between Flex with LSF and RSF *p* ≤ 0.05.

## Data Availability

Not applicable.
